# Nurses Working in and With Adult Protective Services: Agency Characteristics and Job Responsibilities

**DOI:** 10.1093/geroni/igaa057.2437

**Published:** 2020-12-16

**Authors:** Pi-Ju Liu, Jessica Hernandez Chilatra

**Affiliations:** 1 Purdue University, West Lafayette, Indiana, United States; 2 Universidad Nacional de Colombia, Bogotá, Distrito Capital de Bogota, Colombia

## Abstract

The majority of Adult Protective Services (APS) workforce is staffed by social workers, though some agencies have recognized the need to address clients’ medical needs such as wounds, injuries, nutrition issues, hydration issues, premature death and more. Using survey data from the National Adult Protective Services Association (NAPSA), we analyzed 99 nurses’ responses on their role in working in/with APS to help abused, neglect, and exploited adults. Out of the 99 nurses, 65 were direct employees of APS, and 61 did not report directly to a nurse supervisor. Forty-nine nurses carry a caseload like social workers, and 27 carry a caseload in conjunction with social workers. The most common services nurses provide are home visits, evaluations of clients and their medications, and client education. Qualitative data revealed the benefits of having nurses on staff, including assessing medical needs, preventing medical emergencies, providing holistic care, and navigating the healthcare system. Part of a symposium sponsored by Abuse, Neglect and Exploitation of Elderly People Interest Group.

